# Ethyl 3-(4-chloro­phen­yl)-2-phenyl-3-(4-phenyl-1,2,3-selena­diazol-5-yl)propano­ate

**DOI:** 10.1107/S1600536813017790

**Published:** 2013-07-10

**Authors:** P. Sugumar, S. Sankari, P. Manisankar, V. Thiruselvam, M. N. Ponnuswamy

**Affiliations:** a Centre of Advanced Study in Crystallography and Biophysics, University of Madras, Guindy Campus, Chennai 600 025, India; b Department of Chemistry, Sri Sarada College for Women (Autonomous), Fairlands, Salem 636 016, India; c Department of Industrial Chemistry, Alagappa University, Karaikudi 630 003, India

## Abstract

In the title compound, C_25_H_21_ClN_2_O_2_Se, the selena­diazole ring is almost planar [maximum deviation = 0.004 (2) Å], and the adjacent benzene ring is twisted by 50.6 (1)° with respect to this ring.

## Related literature

For general background to selena­diazol derivatives, see: Khanna (2005 ▶). For related structures, see: Marx *et al.* (2008 ▶); Muthukumaran *et al.* (2011 ▶).
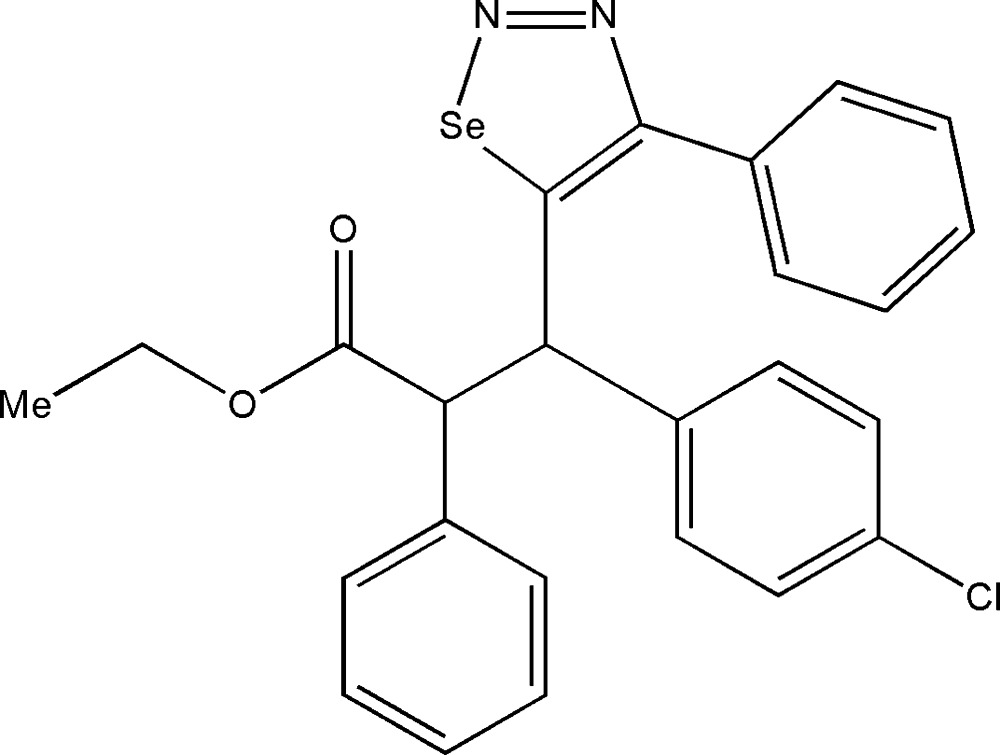


## Experimental

### 

#### Crystal data

C_25_H_21_ClN_2_O_2_Se
*M* *_r_* = 495.85
Monoclinic, 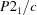

*a* = 12.1337 (3) Å
*b* = 12.2267 (3) Å
*c* = 16.4423 (4) Å
β = 107.744 (1)°
*V* = 2323.26 (10) Å^3^
*Z* = 4
Mo *K*α radiation
μ = 1.76 mm^−1^
*T* = 293 K
0.25 × 0.20 × 0.18 mm

#### Data collection

Bruker SMART APEXII CCD diffractometer
Absorption correction: multi-scan (*SADABS*; Bruker, 2008 ▶) *T*_min_ = 0.663, *T*_max_ = 0.729
22323 measured reflections
5764 independent reflections
3745 reflections with *I* > 2σ(*I*)
*R*_int_ = 0.035

#### Refinement

*R*[*F* ^2^ > 2σ(*F* ^2^)] = 0.037
*wR*(*F* ^2^) = 0.107
*S* = 1.02
5764 reflections
280 parameters
H-atom parameters constrained
Δρ_max_ = 0.42 e Å^−3^
Δρ_min_ = −0.27 e Å^−3^

### 

Data collection: *APEX2* (Bruker, 2008 ▶); cell refinement: *SAINT* (Bruker, 2008 ▶); data reduction: *SAINT*; program(s) used to solve structure: *SHELXS97* (Sheldrick, 2008 ▶); program(s) used to refine structure: *SHELXL97* (Sheldrick, 2008 ▶); molecular graphics: *ORTEP-3 for Windows* (Farrugia, 2012 ▶); software used to prepare material for publication: *SHELXL97* and *PLATON* (Spek, 2009 ▶).

## Supplementary Material

Crystal structure: contains datablock(s) global, I. DOI: 10.1107/S1600536813017790/ng5333sup1.cif

Structure factors: contains datablock(s) I. DOI: 10.1107/S1600536813017790/ng5333Isup2.hkl

Supplementary material file. DOI: 10.1107/S1600536813017790/ng5333Isup3.cml

Additional supplementary materials:  crystallographic information; 3D view; checkCIF report

## Figures and Tables

**Fig. 1.**
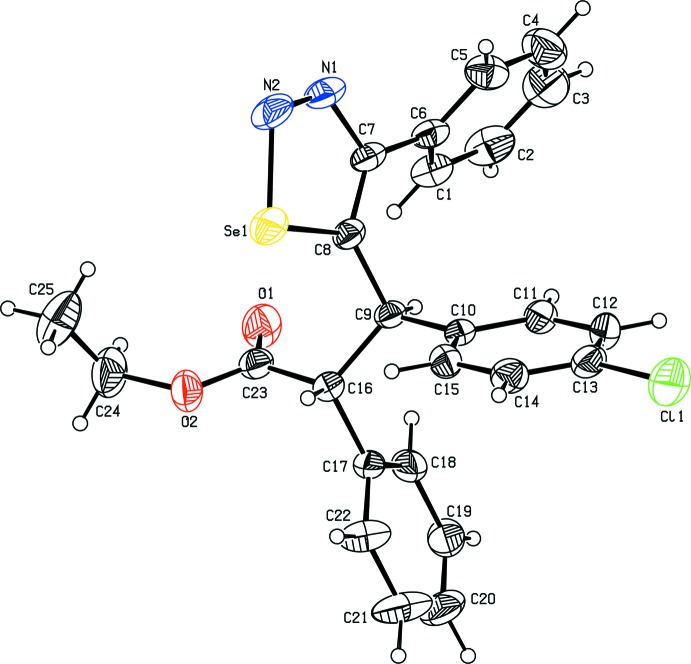
The molecular structure of the title compound with the displacement ellipsoids drawn at 30% probability level.

## supplementary crystallographic information

### Comment

Selenadiazoles, having one selenium and two nitrogen atoms in a five membered ring, are the important class of organoselenium compounds utilized in the synthesis of semiconductor nanoparticles (Khanna, 2005). The crystal structure of the title compound is carried out to elucidate the conformational status of the molecule.

The *ORTEP* plot of the molecule is shown in Fig.1. The selenadiazol ring is planar[with maximum deviation for the atom N2 is -0.004 (2) Å]. The attached phenyl ring is twisted away at an angle of 50.6 (1)° with respect to selenadiazol ring. The bond lengths [Se1—N2] 1.874 (2) Å, [Se1—C8] 1.838 (2) Å & [Cl1—C13] 1.736 (2)° are comparable with the values reported in the literature (Marx *et al.* 2008; Muthukumaran *et al.* 2011). The bond C9—C16 is slightly lengthened due to streic interaction between the phenyl and chlorophenyl rings.

The dihedral angle between the selenadiazol and chlorophenyl ring is 74.3 (1)°. The propanoate group assumes an extended conformation which can be seen from the torsion angle (C16—C23—O2—C24) value of -177.8 (2)°.

### Experimental

A mixture of ethyl 3-(4-chlorophenyl)-5-oxo-2,5-diphenylpentanoate (1 m*M*), semicarbazide hydrochloride(2 m*M*) and sodium acetate (3 m*M*) in ethanol (10 ml) was refluxed for 4 hrs. After completion of the reaction as monitored by TLC, the mixture was poured into ice cold water and the resulting semicarbazone was filtered off. Then, a mixture of semicarbazone (1 m*M*) and SeO_2_ (2 m*M*) in tetrahydrofuran (10 ml) were refluxed on a water bath for 1hr. The selenium deposited on cooling was removed by filtration, and the filtrate was poured into crushed ice, extracted with dichloromethane, and purified by column chromatography using silica gel (60–120 mesh) with 97:3 petroleum ether: ethyl acetate as eluent to give ethyl-3-(4-chlorophenyl)-2-phenyl-3- (4-phenyl-1,2, 3-selenadiazol-5-yl)propanoate.

### Refinement

H atoms were positioned geometrically (C—H = 0.93–0.98 Å) and allowed to ride on their parent atoms, with *U*_iso_(H) = 1.5*U*_eq_(C) for methyl H and 1.2*U*_eq_(C) for other H atoms.

### Crystal data

**Table:**

C_25_H_21_ClN_2_O_2_Se	*F*(000) = 1008
*M**_r_* = 495.85	*D*_x_ = 1.418 Mg m^−^^3^
Monoclinic, *P*2_1_/*c*	Mo *K*α radiation, λ = 0.71073 Å
Hall symbol: -P 2ybc	Cell parameters from 5764 reflections
*a* = 12.1337 (3) Å	θ = 1.8–28.4°
*b* = 12.2267 (3) Å	µ = 1.76 mm^−^^1^
*c* = 16.4423 (4) Å	*T* = 293 K
β = 107.744 (1)°	Block, yellow
*V* = 2323.26 (10) Å^3^	0.25 × 0.20 × 0.18 mm
*Z* = 4	

### Data collection

**Table:**

Bruker SMART APEXII CCD diffractometer	5764 independent reflections
Radiation source: fine-focus sealed tube	3745 reflections with *I* > 2σ(*I*)
Graphite monochromator	*R*_int_ = 0.035
ω and φ scans	θ_max_ = 28.4°, θ_min_ = 1.8°
Absorption correction: multi-scan (*SADABS*; Bruker, 2008)	*h* = −12→16
*T*_min_ = 0.663, *T*_max_ = 0.729	*k* = −16→15
22323 measured reflections	*l* = −21→19

### Refinement

**Table:**

Refinement on *F*^2^	Primary atom site location: structure-invariant direct methods
Least-squares matrix: full	Secondary atom site location: difference Fourier map
*R*[*F*^2^ > 2σ(*F*^2^)] = 0.037	Hydrogen site location: inferred from neighbouring sites
*wR*(*F*^2^) = 0.107	H-atom parameters constrained
*S* = 1.02	*w* = 1/[σ^2^(*F*_o_^2^) + (0.0471*P*)^2^ + 0.6047*P*] where *P* = (*F*_o_^2^ + 2*F*_c_^2^)/3
5764 reflections	(Δ/σ)_max_ = 0.002
280 parameters	Δρ_max_ = 0.42 e Å^−^^3^
0 restraints	Δρ_min_ = −0.27 e Å^−^^3^

### Special details

**Table:**

Geometry. All e.s.d.'s (except the e.s.d. in the dihedral angle between two l.s. planes) are estimated using the full covariance matrix. The cell e.s.d.'s are taken into account individually in the estimation of e.s.d.'s in distances, angles and torsion angles; correlations between e.s.d.'s in cell parameters are only used when they are defined by crystal symmetry. An approximate (isotropic) treatment of cell e.s.d.'s is used for estimating e.s.d.'s involving l.s. planes.
Refinement. Refinement of *F*^2^ against ALL reflections. The weighted *R*-factor *wR* and goodness of fit *S* are based on *F*^2^, conventional *R*-factors *R* are based on *F*, with *F* set to zero for negative *F*^2^. The threshold expression of *F*^2^ > σ(*F*^2^) is used only for calculating *R*-factors(gt) *etc*. and is not relevant to the choice of reflections for refinement. *R*-factors based on *F*^2^ are statistically about twice as large as those based on *F*, and *R*- factors based on ALL data will be even larger.

### Fractional atomic coordinates and isotropic or equivalent isotropic displacement parameters (Å^2^)

**Table:**

	*x*	*y*	*z*	*U*_iso_*/*U*_eq_	
C1	0.7760 (2)	0.5374 (2)	0.74986 (16)	0.0633 (6)	
H1	0.7656	0.5310	0.6916	0.076*	
C2	0.7134 (3)	0.6137 (2)	0.7793 (2)	0.0836 (9)	
H2	0.6610	0.6588	0.7407	0.100*	
C3	0.7278 (3)	0.6234 (3)	0.8652 (3)	0.0954 (10)	
H3	0.6847	0.6744	0.8846	0.114*	
C4	0.8054 (3)	0.5580 (3)	0.9219 (2)	0.0908 (10)	
H4	0.8151	0.5647	0.9800	0.109*	
C5	0.8696 (2)	0.4818 (2)	0.89387 (17)	0.0706 (7)	
H5	0.9230	0.4381	0.9331	0.085*	
C6	0.85467 (19)	0.47046 (18)	0.80734 (14)	0.0536 (5)	
C7	0.92554 (18)	0.38995 (18)	0.77867 (13)	0.0502 (5)	
C8	0.88943 (17)	0.31106 (18)	0.71769 (13)	0.0469 (5)	
C9	0.76586 (16)	0.28344 (17)	0.66740 (12)	0.0428 (4)	
H9	0.7191	0.3492	0.6659	0.051*	
C10	0.72046 (16)	0.19519 (17)	0.71371 (12)	0.0426 (4)	
C11	0.64815 (19)	0.2233 (2)	0.76108 (15)	0.0549 (5)	
H11	0.6269	0.2960	0.7634	0.066*	
C12	0.6070 (2)	0.1451 (2)	0.80495 (15)	0.0656 (7)	
H12	0.5585	0.1648	0.8367	0.079*	
C13	0.6383 (2)	0.0383 (2)	0.80109 (15)	0.0631 (7)	
C14	0.7116 (2)	0.0082 (2)	0.75615 (15)	0.0622 (6)	
H14	0.7338	−0.0644	0.7551	0.075*	
C15	0.7521 (2)	0.08659 (19)	0.71257 (14)	0.0538 (5)	
H15	0.8016	0.0663	0.6818	0.065*	
C16	0.75310 (17)	0.25174 (17)	0.57420 (13)	0.0456 (5)	
H16	0.7931	0.1820	0.5747	0.055*	
C17	0.62689 (19)	0.23681 (19)	0.52183 (13)	0.0497 (5)	
C18	0.5471 (2)	0.3186 (2)	0.51442 (14)	0.0640 (6)	
H18	0.5699	0.3853	0.5415	0.077*	
C19	0.4325 (2)	0.3023 (3)	0.46674 (16)	0.0834 (9)	
H19	0.3785	0.3579	0.4619	0.100*	
C20	0.3993 (3)	0.2040 (4)	0.4268 (2)	0.0985 (12)	
H20	0.3222	0.1923	0.3958	0.118*	
C21	0.4772 (3)	0.1247 (3)	0.4322 (2)	0.1093 (13)	
H21	0.4542	0.0589	0.4037	0.131*	
C22	0.5919 (3)	0.1399 (2)	0.47981 (18)	0.0818 (8)	
H22	0.6454	0.0842	0.4833	0.098*	
C23	0.80904 (19)	0.3363 (2)	0.53277 (14)	0.0533 (5)	
C24	0.9294 (3)	0.3559 (3)	0.4427 (2)	0.0910 (9)	
H24A	0.9033	0.3406	0.3820	0.109*	
H24B	0.9149	0.4325	0.4509	0.109*	
C25	1.0522 (3)	0.3337 (3)	0.4767 (3)	0.1152 (13)	
H25A	1.0935	0.3782	0.4477	0.173*	
H25B	1.0663	0.2579	0.4683	0.173*	
H25C	1.0781	0.3502	0.5366	0.173*	
N1	1.04418 (17)	0.39168 (19)	0.81995 (13)	0.0663 (5)	
N2	1.10574 (18)	0.3221 (2)	0.79702 (15)	0.0766 (6)	
O1	0.80184 (17)	0.43304 (16)	0.54165 (12)	0.0777 (5)	
O2	0.86582 (16)	0.28857 (15)	0.48558 (11)	0.0679 (5)	
Cl1	0.58491 (9)	−0.06117 (8)	0.85433 (6)	0.1057 (3)	
Se1	1.01461 (2)	0.23132 (3)	0.710678 (17)	0.06928 (12)	

### Atomic displacement parameters (Å^2^)

**Table:**

	*U*^11^	*U*^22^	*U*^33^	*U*^12^	*U*^13^	*U*^23^
C1	0.0524 (13)	0.0566 (14)	0.0712 (15)	−0.0052 (11)	0.0043 (11)	−0.0039 (12)
C2	0.0652 (17)	0.0621 (17)	0.109 (2)	0.0021 (14)	0.0058 (16)	−0.0044 (16)
C3	0.090 (2)	0.079 (2)	0.120 (3)	0.0026 (18)	0.036 (2)	−0.025 (2)
C4	0.119 (3)	0.079 (2)	0.080 (2)	−0.015 (2)	0.039 (2)	−0.0217 (17)
C5	0.0822 (19)	0.0585 (16)	0.0642 (16)	−0.0078 (13)	0.0121 (13)	−0.0058 (12)
C6	0.0494 (12)	0.0467 (12)	0.0576 (13)	−0.0146 (10)	0.0057 (10)	−0.0040 (10)
C7	0.0385 (11)	0.0530 (13)	0.0515 (12)	−0.0106 (9)	0.0025 (9)	0.0027 (10)
C8	0.0350 (10)	0.0535 (12)	0.0485 (11)	−0.0031 (9)	0.0071 (8)	0.0028 (9)
C9	0.0331 (9)	0.0476 (12)	0.0437 (11)	−0.0031 (8)	0.0056 (8)	−0.0046 (8)
C10	0.0336 (9)	0.0515 (12)	0.0391 (10)	−0.0046 (8)	0.0058 (8)	−0.0060 (8)
C11	0.0436 (11)	0.0644 (14)	0.0559 (13)	0.0018 (10)	0.0142 (10)	−0.0053 (11)
C12	0.0503 (13)	0.093 (2)	0.0591 (14)	−0.0091 (13)	0.0247 (11)	−0.0038 (13)
C13	0.0588 (14)	0.0749 (18)	0.0536 (13)	−0.0249 (13)	0.0141 (11)	0.0025 (12)
C14	0.0702 (16)	0.0545 (14)	0.0609 (14)	−0.0117 (12)	0.0185 (12)	−0.0019 (11)
C15	0.0561 (13)	0.0543 (14)	0.0546 (13)	−0.0043 (10)	0.0222 (10)	−0.0054 (10)
C16	0.0383 (10)	0.0516 (13)	0.0451 (11)	−0.0037 (9)	0.0098 (8)	−0.0024 (9)
C17	0.0447 (11)	0.0649 (14)	0.0365 (10)	−0.0117 (10)	0.0082 (8)	−0.0027 (9)
C18	0.0468 (13)	0.0940 (19)	0.0471 (13)	0.0022 (13)	0.0082 (10)	−0.0126 (12)
C19	0.0429 (13)	0.148 (3)	0.0523 (14)	0.0061 (16)	0.0045 (11)	−0.0008 (16)
C20	0.0558 (18)	0.154 (4)	0.0665 (18)	−0.038 (2)	−0.0093 (14)	0.011 (2)
C21	0.097 (3)	0.095 (3)	0.101 (2)	−0.043 (2)	−0.024 (2)	−0.007 (2)
C22	0.0739 (18)	0.0680 (18)	0.0809 (18)	−0.0149 (14)	−0.0103 (14)	−0.0079 (14)
C23	0.0448 (12)	0.0626 (16)	0.0478 (12)	−0.0083 (10)	0.0073 (9)	0.0007 (10)
C24	0.085 (2)	0.114 (3)	0.086 (2)	−0.0062 (19)	0.0439 (17)	0.0262 (18)
C25	0.087 (3)	0.094 (3)	0.179 (4)	−0.016 (2)	0.062 (3)	0.019 (2)
N1	0.0416 (11)	0.0765 (14)	0.0671 (13)	−0.0124 (10)	−0.0039 (9)	−0.0003 (10)
N2	0.0349 (10)	0.0998 (18)	0.0827 (15)	−0.0066 (11)	−0.0002 (10)	−0.0018 (13)
O1	0.0910 (14)	0.0613 (12)	0.0898 (13)	−0.0124 (10)	0.0409 (11)	0.0029 (9)
O2	0.0688 (11)	0.0807 (12)	0.0641 (10)	−0.0032 (9)	0.0352 (9)	0.0054 (9)
Cl1	0.1182 (7)	0.1091 (7)	0.1002 (6)	−0.0457 (5)	0.0485 (5)	0.0149 (5)
Se1	0.03892 (14)	0.0867 (2)	0.0777 (2)	0.00811 (12)	0.01107 (11)	−0.00725 (13)

### Geometric parameters (Å, º)

**Table:**

C1—C2	1.380 (4)	C14—H14	0.9300
C1—C6	1.387 (3)	C15—H15	0.9300
C1—H1	0.9300	C16—C23	1.509 (3)
C2—C3	1.374 (4)	C16—C17	1.522 (3)
C2—H2	0.9300	C16—H16	0.9800
C3—C4	1.364 (5)	C17—C18	1.372 (3)
C3—H3	0.9300	C17—C22	1.372 (3)
C4—C5	1.381 (4)	C18—C19	1.386 (3)
C4—H4	0.9300	C18—H18	0.9300
C5—C6	1.385 (3)	C19—C20	1.371 (5)
C5—H5	0.9300	C19—H19	0.9300
C6—C7	1.476 (3)	C20—C21	1.338 (5)
C7—C8	1.364 (3)	C20—H20	0.9300
C7—N1	1.392 (3)	C21—C22	1.386 (4)
C8—C9	1.513 (3)	C21—H21	0.9300
C8—Se1	1.838 (2)	C22—H22	0.9300
C9—C10	1.518 (3)	C23—O1	1.198 (3)
C9—C16	1.542 (3)	C23—O2	1.320 (3)
C9—H9	0.9800	C24—C25	1.448 (5)
C10—C11	1.382 (3)	C24—O2	1.449 (3)
C10—C15	1.384 (3)	C24—H24A	0.9700
C11—C12	1.379 (3)	C24—H24B	0.9700
C11—H11	0.9300	C25—H25A	0.9600
C12—C13	1.367 (4)	C25—H25B	0.9600
C12—H12	0.9300	C25—H25C	0.9600
C13—C14	1.368 (3)	N1—N2	1.263 (3)
C13—Cl1	1.736 (2)	N2—Se1	1.874 (2)
C14—C15	1.374 (3)		
			
C2—C1—C6	119.8 (3)	C14—C15—H15	119.4
C2—C1—H1	120.1	C10—C15—H15	119.4
C6—C1—H1	120.1	C23—C16—C17	109.90 (17)
C3—C2—C1	120.5 (3)	C23—C16—C9	110.70 (17)
C3—C2—H2	119.7	C17—C16—C9	111.81 (17)
C1—C2—H2	119.7	C23—C16—H16	108.1
C4—C3—C2	119.8 (3)	C17—C16—H16	108.1
C4—C3—H3	120.1	C9—C16—H16	108.1
C2—C3—H3	120.1	C18—C17—C22	118.9 (2)
C3—C4—C5	120.6 (3)	C18—C17—C16	121.6 (2)
C3—C4—H4	119.7	C22—C17—C16	119.5 (2)
C5—C4—H4	119.7	C17—C18—C19	120.3 (3)
C4—C5—C6	120.0 (3)	C17—C18—H18	119.8
C4—C5—H5	120.0	C19—C18—H18	119.8
C6—C5—H5	120.0	C20—C19—C18	119.7 (3)
C5—C6—C1	119.2 (2)	C20—C19—H19	120.2
C5—C6—C7	119.2 (2)	C18—C19—H19	120.2
C1—C6—C7	121.5 (2)	C21—C20—C19	120.3 (3)
C8—C7—N1	115.0 (2)	C21—C20—H20	119.8
C8—C7—C6	128.22 (19)	C19—C20—H20	119.8
N1—C7—C6	116.80 (19)	C20—C21—C22	120.6 (3)
C7—C8—C9	127.0 (2)	C20—C21—H21	119.7
C7—C8—Se1	109.56 (15)	C22—C21—H21	119.7
C9—C8—Se1	123.19 (16)	C17—C22—C21	120.2 (3)
C8—C9—C10	109.59 (16)	C17—C22—H22	119.9
C8—C9—C16	112.45 (17)	C21—C22—H22	119.9
C10—C9—C16	112.21 (16)	O1—C23—O2	125.4 (2)
C8—C9—H9	107.4	O1—C23—C16	124.2 (2)
C10—C9—H9	107.4	O2—C23—C16	110.4 (2)
C16—C9—H9	107.4	C25—C24—O2	110.1 (3)
C11—C10—C15	118.2 (2)	C25—C24—H24A	109.6
C11—C10—C9	119.7 (2)	O2—C24—H24A	109.6
C15—C10—C9	122.06 (19)	C25—C24—H24B	109.6
C12—C11—C10	121.1 (2)	O2—C24—H24B	109.6
C12—C11—H11	119.5	H24A—C24—H24B	108.2
C10—C11—H11	119.5	C24—C25—H25A	109.5
C13—C12—C11	119.1 (2)	C24—C25—H25B	109.5
C13—C12—H12	120.4	H25A—C25—H25B	109.5
C11—C12—H12	120.4	C24—C25—H25C	109.5
C14—C13—C12	121.2 (2)	H25A—C25—H25C	109.5
C14—C13—Cl1	119.2 (2)	H25B—C25—H25C	109.5
C12—C13—Cl1	119.6 (2)	N2—N1—C7	117.5 (2)
C13—C14—C15	119.3 (2)	N1—N2—Se1	111.03 (15)
C13—C14—H14	120.4	C23—O2—C24	119.0 (2)
C15—C14—H14	120.4	C8—Se1—N2	86.93 (10)
C14—C15—C10	121.2 (2)		
			
C6—C1—C2—C3	−0.4 (4)	C11—C10—C15—C14	1.0 (3)
C1—C2—C3—C4	0.7 (5)	C9—C10—C15—C14	178.84 (19)
C2—C3—C4—C5	−0.1 (5)	C8—C9—C16—C23	−50.6 (2)
C3—C4—C5—C6	−0.7 (5)	C10—C9—C16—C23	−174.72 (17)
C4—C5—C6—C1	1.0 (4)	C8—C9—C16—C17	−173.52 (18)
C4—C5—C6—C7	179.0 (2)	C10—C9—C16—C17	62.4 (2)
C2—C1—C6—C5	−0.5 (4)	C23—C16—C17—C18	−66.8 (3)
C2—C1—C6—C7	−178.4 (2)	C9—C16—C17—C18	56.6 (3)
C5—C6—C7—C8	129.5 (3)	C23—C16—C17—C22	112.2 (3)
C1—C6—C7—C8	−52.6 (3)	C9—C16—C17—C22	−124.4 (2)
C5—C6—C7—N1	−48.6 (3)	C22—C17—C18—C19	1.4 (4)
C1—C6—C7—N1	129.3 (2)	C16—C17—C18—C19	−179.6 (2)
N1—C7—C8—C9	174.8 (2)	C17—C18—C19—C20	−0.1 (4)
C6—C7—C8—C9	−3.3 (4)	C18—C19—C20—C21	−1.4 (5)
N1—C7—C8—Se1	−0.1 (2)	C19—C20—C21—C22	1.6 (6)
C6—C7—C8—Se1	−178.24 (18)	C18—C17—C22—C21	−1.3 (4)
C7—C8—C9—C10	−91.3 (3)	C16—C17—C22—C21	179.7 (3)
Se1—C8—C9—C10	83.0 (2)	C20—C21—C22—C17	−0.2 (6)
C7—C8—C9—C16	143.2 (2)	C17—C16—C23—O1	82.5 (3)
Se1—C8—C9—C16	−42.5 (2)	C9—C16—C23—O1	−41.5 (3)
C8—C9—C10—C11	101.5 (2)	C17—C16—C23—O2	−97.2 (2)
C16—C9—C10—C11	−132.8 (2)	C9—C16—C23—O2	138.78 (19)
C8—C9—C10—C15	−76.3 (2)	C8—C7—N1—N2	0.5 (3)
C16—C9—C10—C15	49.4 (2)	C6—C7—N1—N2	178.9 (2)
C15—C10—C11—C12	−1.1 (3)	C7—N1—N2—Se1	−0.7 (3)
C9—C10—C11—C12	−178.98 (19)	O1—C23—O2—C24	2.6 (4)
C10—C11—C12—C13	−0.1 (4)	C16—C23—O2—C24	−177.8 (2)
C11—C12—C13—C14	1.5 (4)	C25—C24—O2—C23	115.2 (3)
C11—C12—C13—Cl1	−178.78 (18)	C7—C8—Se1—N2	−0.22 (17)
C12—C13—C14—C15	−1.5 (4)	C9—C8—Se1—N2	−175.37 (18)
Cl1—C13—C14—C15	178.69 (18)	N1—N2—Se1—C8	0.5 (2)
C13—C14—C15—C10	0.3 (3)		
